# Dynamics of Intact and Defective Human Immunodeficiency Virus Type 1 (HIV-1) Proviruses During Decades of Suppressive Antiretroviral Treatment in Young Adults With Perinatal HIV

**DOI:** 10.1093/infdis/jiag045

**Published:** 2026-01-31

**Authors:** Priya Khetan, Kunjal Patel, Wendy Yu, Joseph Szewczyk, Adit Dhummakupt, Sandra Burchett, Russell B Van Dyke, Deborah Persaud

**Affiliations:** Department of Pediatrics, Division of Infectious Diseases, Johns Hopkins University School of Medicine, Baltimore, Maryland, USA; Department of Epidemiology, Harvard T.H. Chan School of Public Health, Boston, Massachusetts, USA; Center for Biostatistics in AIDS Research, Harvard T.H. Chan School of Public Health, Boston, Massachusetts, USA; Center for Biostatistics in AIDS Research, Harvard T.H. Chan School of Public Health, Boston, Massachusetts, USA; Department of Pediatrics, Division of Infectious Diseases, Johns Hopkins University School of Medicine, Baltimore, Maryland, USA; Department of Pediatrics, Division of Infectious Diseases, Johns Hopkins University School of Medicine, Baltimore, Maryland, USA; Department of Pediatrics, Division of Infectious Diseases, Boston Children's Hospital, Boston, Massachusetts, USA; Department of Pediatrics, Section of Infectious Diseases, Tulane University School of Medicine, New Orleans, Louisiana, USA; Department of Pediatrics, Division of Infectious Diseases, Johns Hopkins University School of Medicine, Baltimore, Maryland, USA

**Keywords:** proviral reservoir, HIV, perinatal, young adults

## Abstract

**Background:**

Understanding HIV-1 reservoir dynamics during long-term antiretroviral therapy (ART) in youth with perinatal Human Immunodeficiency Virus Type-1 (HIV-1) is essential for ART-free remission strategies.

**Methods:**

We quantified intact and defective HIV proviruses in 201 peripheral blood mononuclear cell samples (PBMCs) from participants ages 17.6–21.2 years in the Pediatric HIV/AIDS Cohort Study (PHACS) Adolescent Master Protocol. Participants were classified as early-suppressed (ES, <1 year of age at virologic suppression [VS]) or late-suppressed (LS, 1–5 years of age at VS) and had maintained VS for up to 20 years.

**Results:**

Twenty-six participants (11 ES and 15 LS) were evaluated. Early-suppressed participants exhibited significantly lower intact HIV reservoirs compared with LS participants, with 67% of ES samples below detection limits (2.0 copies/10^6^ PBMCs). By 5 years of VS, the ES participants had significantly lower mean intact proviral load (2.0 vs 6.6 copies/10^6^ PBMCs) than LS participants, largely driven by faster clearance of intact proviruses in the first 5 years of VS. Among LS participants, females had larger intact reservoirs than males (mean: 12.5 vs 4.1 intact copies/10^6^ PBMCs) and exhibited greater increases in defective proviruses over time.

**Conclusions:**

Achieving VS by 1 year of age in perinatal HIV infection results in substantially smaller HIV intact reservoirs by age 5, with effects sustained through young adulthood. Additionally, sex-based differences, larger intact reservoirs and increases in defective proviruses in females, underscore the need for tailored ART-free remission and cure strategies for this population.

## Teaser

In perinatal HIV, early age to virologic suppression impacts the decay pattern and size of the intact proviral reservoir into young adulthood. With late virologic suppression, females had larger proviral loads than males, highlighting sex differences in perinatal HIV.

## Graphical Abstract

**Graphical Abstract:**
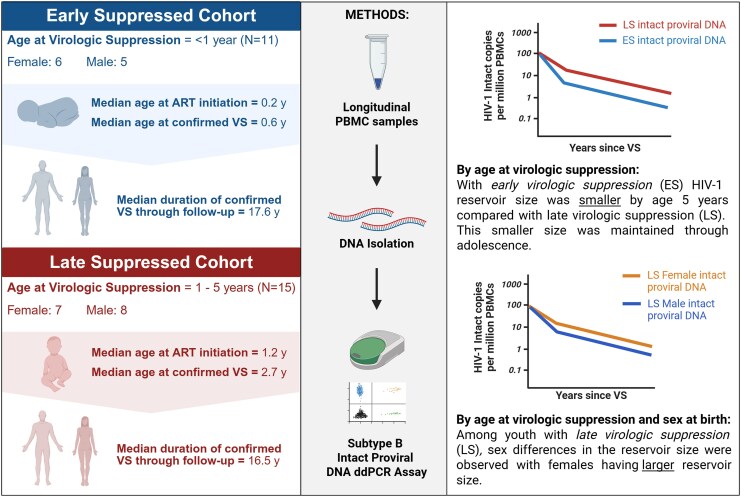

**(See the Editorial Commentary by Gandhi et al on pages e1310–2.)**

An estimated 1.4 million children worldwide are living with perinatal Human Immunodeficiency Virus Type-1 (HIV-1, PHIV) [1] and face a lifetime of antiretroviral therapy (ART) due to the early establishment of HIV latency in resting memory CD4+ T cells [2–4]. This latent intact proviral reservoir is not targeted by ART regimens and retains replication competence, capable of refueling viremic rebound and preventing ART discontinuation [5–7]. A recent study in adults with a mean of 22 years on ART showed that while the latent HIV reservoir decays slowly over the first 7 years of ART (T_1/2_ = 44 months), the reservoir expands, likely from clonal expansion of CD4+ T cells [8]. Understanding HIV reservoir dynamics during ART in children and young adults with perinatal HIV is critical for assessing remission strategies for this population.

Single-plex HIV DNA quantitative assays have been the main biomarker used to measure HIV reservoir size in children receiving ART, providing insights into how early ART initiation and virologic suppression (VS) in infancy compared with delayed VS in later childhood leads to smaller reservoir size [9]. However, more than 90% of proviral genomes are defective due to large deletions, insertions, reversions, and hypermutations [10]. Thus, single-plex assays have grossly overestimated the reservoir size. Accurate measurements of reservoir size are becoming critical for evaluating efficacy of HIV treatments aimed at reservoir clearance and cure [11]. A newly developed HIV DNA assay, the intact proviral DNA assay (IPDA), enables approximation of intact and defective proviruses within the total HIV DNA pool [12]. Recently developed non-subtype B-specific IPDAs have allowed broader applications for studies of HIV reservoirs worldwide, where HIV subtypes differ [13–15].

Using a single-plex HIV DNA assay, we previously studied HIV reservoir dynamics in US adolescents with subtype B PHIV as part of the Adolescent Master Protocol (AMP) conducted by the Pediatric HIV/AIDS Cohort Study (PHACS). We found that adolescents who initiated ART in early infancy and achieved VS by 1 year of age (early-suppressed [ES]) had significantly lower total HIV DNA loads at 5 and 10 years of VS compared to those who achieved VS in later childhood between 1 and 5 years of age (late-suppressed [LS]) [16]. Similar associations between earlier VS and smaller reservoirs in cohorts of children with non-subtype B HIV infections were reported [9], highlighting the impact of age at VS on shaping PHIV reservoir dynamics.

In this study, we applied the subtype B, multiplex HIV IPDA to the same PHACS Cohort of ES and LS participants to characterize HIV reservoir dynamics more precisely through analyses of the trajectories of intact and defective proviruses over up to 20 years of VS. We also evaluated differences between males and females, given known sex-based variations in CD4+ T-cell dynamics.

## METHODS

### Study Population

Participants were from the PHACS AMP study, a US-based prospective cohort study of the long-term outcomes of children and adolescents with PHIV. We identified participants in PHACS AMP who initiated ART, achieved VS by 5 years of age, maintained VS through their last study visit in AMP or AMP Up, the follow-up study to AMP before 1 April 2020, and had available peripheral blood mononuclear cells (PBMCs) during VS. Early-suppressed participants achieved VS by 1 year of age and LS participants between 1 and 5 years of age. We defined ART as a regimen of at least 3 drugs from at least 2 antiretroviral drug classes. We defined initial VS as 2 consecutive HIV RNA viral loads <400 copies/mL after ART initiation. Maintaining VS was defined as maintaining HIV RNA viral loads <400 copies/mL, allowing for isolated measures ≥400 copies/mL between measures <400 copies/mL (ie, viral blips) [16]. There were no upper limits to the copies/mL or number of blips detected. To minimize the possibility of low-level viral replication and ensure that included participants truly maintained long-term VS, we excluded those with measured levels of 2-LTR circles ≥10 copies/million PBMCs at 2 or more years after VS from a previous study. We matched LS participants to ES participants by age at the last available PBMC sample, percent without viral blips, and sex.

The study received ethical approval from the Harvard T.H. Chan School of Public Health's institutional review board (IRB) and from the IRBs of each clinical research site. Written informed consent was received from all participants before inclusion in the study.

### Genomic DNA Isolation

We isolated genomic DNA from 5 million PBMCs using the Qiagen Puregene Cell Kit (Qiagen, Hilden, Germany) with these modifications: PBMCs were lysed overnight at room temperature, and DNA was allowed to dissolve in 50 µL of DNA hydration solution overnight at 25°C.

### HIV DNA Quantification

Intact (detection of both the Ψ and env region), 5′ defective (detection of the env region only), and 3′ defective/hypermutated (detection of the Ψ region only) HIV DNA copies/million PBMCs were quantified using the IPDA described in previous publications [12]. Samples were run with 200 ng input of genomic DNA per well in 16 replicates. We determined the number of cells analyzed and percent of unsheared genomic DNA, using primers targeting 2 regions of the housekeeping gene RPP30. The assay's limit of detection (LOD) for each PBMC sample was determined by the number of PBMCs available for analysis. Undetected values were imputed as half of the LOD for analyses. Total proviral HIV DNA load was the sum of intact, 5′ defective, and 3′ defective/hypermutated HIV DNA after imputation.

### Statistical Methods

The study population's demographic, ART, virologic, and immunologic characteristics were summarized by age at VS with descriptive statistics. Intact, 5′ defective, 3′ defective/hypermutated, and total proviral loads among all PBMC samples analyzed during VS were described by age at VS, biological sex, and VS follow-up period (0 to <5 years, 5 to <10 years, 10 to <15 years, and 15 to <20 years of VS). Locally estimated scatterplot smoothing (LOESS) plots were used to graphically explore trajectories of each proviral HIV DNA over VS by age at VS and sex. We used LOESS plots of total HIV DNA by age at VS in piecewise linear mixed effects regression models to inform knot placement at 5 years of VS. Piecewise linear mixed effects models, including random intercept and random slopes, were fit to estimate trajectories of each log_10_ transformed proviral HIV DNA from 0 to 5 years of VS and from 5–15 years of VS by age at VS. These models were also used to estimate mean concentrations of intact, 5′ defective, 3′ defective/hypermutated and total proviral loads at VS, 5 years, and 15 years of VS. To explore sex differences, models were fit for each proviral HIV1 DNA by sex, stratified by age at VS.

To account for potential dilutional effects of CD4+ T-cell reconstitution following VS on HIV-infected cell concentrations, HIV DNA concentrations were normalized for the number of CD4+ T cells. This was done by dividing PBMC-associated HIV DNA by the proportion of PBMCs that were CD4+ T cells. All analyses were conducted using SAS software, version 9.4 (SAS Institute). We stratified by age at VS and explored the relationship between sex and HIV reservoir size over time since VS.

## RESULTS

### Study Population

Of the 451 participants with PHIV enrolled in the PHACS AMP study, 36 were ES and 163 were LS; 33% (12/36) of ES participants and 27% (44/163) of LS participants maintained VS throughout observed follow-up and had PBMCs available for study. Late-suppressed participants were matched to ES participants by the duration and extent of VS and sex assigned at birth. Transient episodes of detectable viremia during VS were allowed (≥400 copies/mL between measures, ie, viral blips). One ES participant was excluded from the analysis due to the IPDA primers' failure to amplify HIV DNA. The final study population included 11 ES participants (6 females, 5 males) and 15 LS participants (7 females, 8 males).

Table 1 summarizes the study population's demographic, ART, virologic, and immunologic characteristics by age at VS (ie, ES or LS). The distribution year of birth was similar for ES and LS participants (median [IQR]: 1999 [1997–2000] for ES and 1998 [1996–2000] for LS). Most participants received a protease inhibitor-based regimen for initial ART. The ES and LS participants' median ages at ART initiation were 1.9 months and 1.2 years, respectively. The median time from ART initiation to VS was 4.2 months and 8.9 months. Most samples were from 5 to 15 years of VS, limiting interpretation of findings between 1–5 and 15–20 years of VS. We analyzed 201 longitudinal PBMC samples (84 for ES and 117 for LS participants).

**Table 1.** Demographic, ART, Virologic, and Immunologic Characteristics by Age at Virologic Suppression

Characteristic^a^	Total (N = 26)	Early Suppressed <1 Year Old (N = 11)	Late Suppressed 1–5 Years Old (N = 15)
Female sex at birth	13 (50%)	6 (55%)	7 (47%)
Self-reported Black race	23 (88%)	11 (100%)	12 (80%)
Year of birth	1999 (1997, 2000)	1999 (1997, 2000)	1998 (1996, 2000)
Received antiretrovirals as treatment before ART	11 (42%)	3 (27%)	8 (53%)
Age at ART initiation (months)	4.6 (1.8, 15.0)	1.9 (1.6, 3.6)	14.8 (3.8, 20.7)
HIV1 RNA at ART initiation^b^ (log_10_ c/mL)	5.4 (4.6, 5.7)	5.6 (3.9, 6.3)	5.0 (4.8, 5.4)
Missing (%)	2 (8%)	0 (0%)	2 (13%)
CD4% at ART initiation^b^	33 (23, 46)	32 (26, 36)	38 (19, 47)
Missing (%)	4 (15%)	1 (9%)	3 (20%)
1st ART regimen initiated			
PI + NNRTI-based	3 (12%)	2 (18%)	1 (7%)
PI-based	21 (81%)	8 (73%)	13 (87%)
NNRTI-based	2 (8%)	1 (9%)	1 (7%)
Age at virologic suppression (months)	14.4 (8.0, 34.0)	7.8 (5.7, 9.8)	31.8 (19.2, 43.7)
Time from ART initiation to virologic suppression (months)	6.2 (4.2, 17.4)	4.2 (3.7, 7.1)	8.9 (5.1, 34.6)
Time from ART initiation to end of follow-up^c^ (years)	18.2 (16.8, 20.4)	18.2 (16.8, 20.4)	18.7 (15.9, 20.4)
Time from virologic suppression to end of follow-up^c^ (years)	17.3 (15.5, 19.1)	17.6 (16.5, 20.2)	16.5 (14.8, 19.1)
Age at end of follow-up^c^ (years)	18.6 (17.6, 21.2)	18.5 (16.9, 20.9)	18.9 (17.6, 21.5)
CD4 count at end of follow-up^c,d^	771 (522, 909)	793 (509, 889)	682 (522, 1380)
Missing (%)	1 (4%)	1 (9%)	0 (0%)
CD4% at end of follow-up^c,d^	42 (37, 46)	40 (36, 45)	42 (38, 46)
Missing (%)	1 (4%)	1 (9%)	0 (0%)
Average number HIV RNA readings per year during virologic suppression^e^	3.7 (3.1, 4.0)	4.0 (3.2, 4.8)	3.7 (2.7, 3.8)
HIV RNA readings ≥400 c/mL during virologic suppression^e^ (%)	0.7 (0.0, 2.3)	1.3 (0.0, 2.3)	0.0 (0.0, 2.6)
PBMC samples per participant during virologic suppression^e^	8 (6, 10)	9 (5, 12)	8 (7, 10)

Abbreviations: ART, antiretroviral treatment; NNRTI, non-nucleoside reverse transcriptase inhibitor; PBMC, peripheral blood mononuclear cell; PI, protease inhibitor.

^a^Summary statistics presented as either median (Q1, Q3) or N (%).

^b^Closest reading within 6 months preceding ART initiation.

^c^End of follow-up defined as date of last PBMC specimen analyzed.

^d^Closest reading within period ±3 months of last PBMC specimen.

^e^Follow-up time spans from confirmed virologic suppression through to last PBMC specimen analyzed.

### Proportion of Samples With Detectable Intact Proviruses

Overall, a median of 511 836 PBMCs were analyzed per sample from the participants. Of 15 PBMC samples from ES participants obtained during the first 5 years of VS, 33% had measurable intact proviruses compared with 89% of 18 samples among LS participants. From 5 to 15 years of VS, 33% of 69 PBMC samples from ES participants had detectable intact proviruses compared with 73% of 99 samples from LS participants (Table 2).

**Table 2.** HIV Proviral DNA Among All PBMC Samples Analyzed by Follow-Up Period for Participants in the Study Population by Age at Virologic Suppression

	Early Suppressed <1 Year Old (N = 11)				Late Suppressed 1–5 Years Old (N = 15)			
Duration of VS (years)	0 to <5	5 to <10	10 to <15	15 to <20	0 to <5	5 to <10	10 to <15	15 to <20
# of participants studied	6	10	10	10	7	14	15	10
# of samples analyzed	15	22	25	22	18	31	51	17
Intact								
HIV DNA cpm PBMCs	1.0 (0.9, 15.8)	1.2 (0.9, 3.1)	1.1 (1.0, 2.9)	1.1 (0.8, 1.9)	24.8 (4.1, 60.2)	6.4 (3.0, 18.3)	6.3 (1.0, 14.5)	3.8 (1.1, 11.5)
# of detectable samples (%)	5 (33%)	7 (32%)	8 (32%)	8 (36%)	16 (89%)	25 (81%)	35 (69%)	12 (71%)
5′ defective								
HIV DNA cpm PBMCs	3.9 (2.3, 6.6)	9.0 (2.4, 16.4)	10.5 (5.7, 17.6)	9.4 (5.4, 27.4)	36.7 (17.2, 86.0)	34.7 (21.0, 65.6)	19.6 (7.3, 54.9)	16.4 (4.4, 45.8)
# of detectable samples (%)	12 (80%)	20 (91%)	25 (100%)	20 (91%)	16 (89%)	31 (100%)	46 (90%)	15 (88%)
3′ defective/hypermutated								
HIV DNA cpm PBMCs	4.9 (1.1, 11.6)	7.3 (3.6, 15.4)	16.5 (6.4, 29.0)	9.2 (5.0, 14.5)	52.3 (28.1, 112.5)	57.6 (34.7, 137.9)	56.8 (24.2, 121.1)	27.4 (19.1, 88.1)
# of detectable samples (%)	11 (73%)	18 (82%)	23 (92%)	22 (100%)	18 (100%)	31 (100%)	50 (98%)	17 (100%)
Total								
HIV DNA cpm PBMCs	17.3 (12.2, 39.4)	20.0 (8.1, 32.9)	28.0 (18.8, 53.1)	19.7 (13.0, 44.5)	157.8 (92.0, 235.8)	113.2 (68.3, 204.4)	103.3 (47.0, 188.8)	39.9 (25.1, 145.5)
# of detectable samples (%)	15 (100%)	21 (95%)	25 (100%)	22 (100%)	18 (100%)	31 (100%)	51 (100%)	17 (100%)

### Dynamics of Intact Proviruses

**Figure 1.**
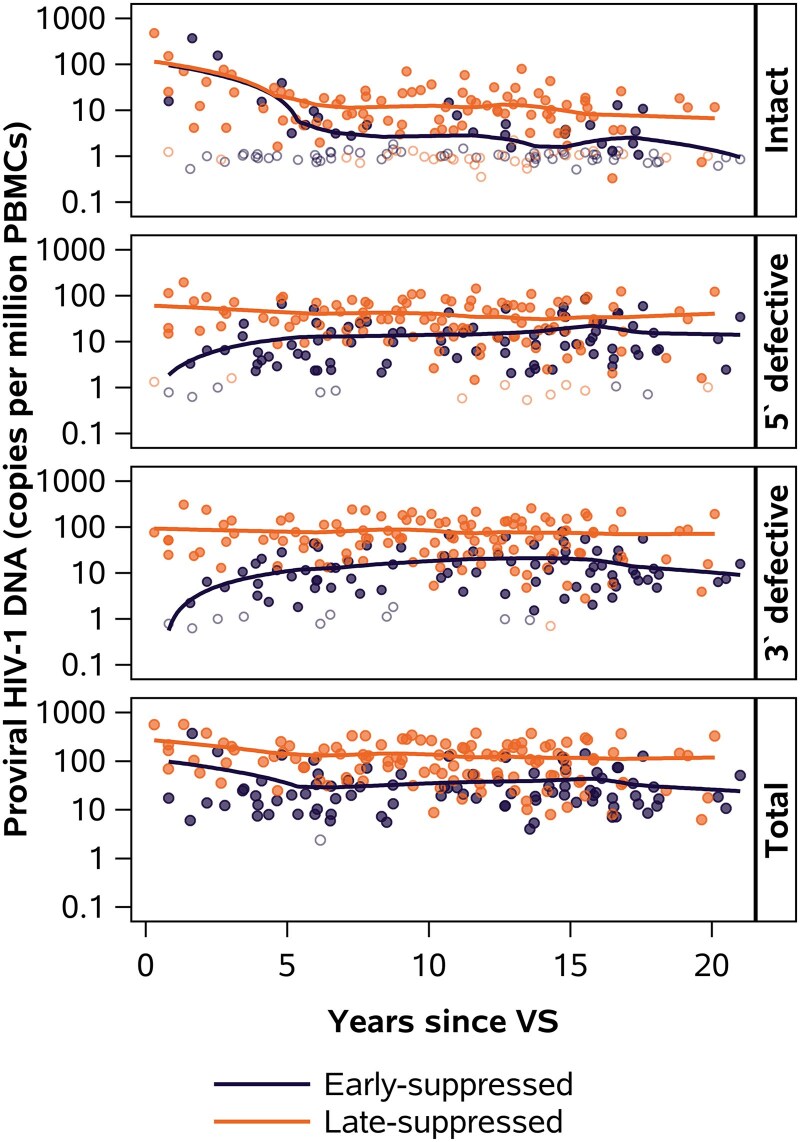
LOESS of intact, 5′ defective, 3′ defective/hypermutated, and total HIV DNA during virologic suppression by age at virologic suppression. Figure presents proviral HIV DNA concentration in copies/million PBMCs by years since VS, with LOESS trend lines overlaid atop actual data points. Unfilled circles represent values below the assay limit of detection which were imputed to be half the limit of detection. Abbreviations: PBMCs, peripheral blood mononuclear cells; VS, virologic suppression.

The LOESS curve for ES and LS participant samples showed a sharp decline in intact proviruses during the first 5 years of VS, followed by a more gradual decline from 5 to 20 years of VS (Figure 1). The median intact HIV DNA level from 5 to 10 years and 10 to 15 years of VS was 1.2 copies/million and 1.1 copies/million PBMCs in ES versus 6.4 and 6.3 copies/million PBMCs in LS participants **(**Table 2).

**Figure 2.**
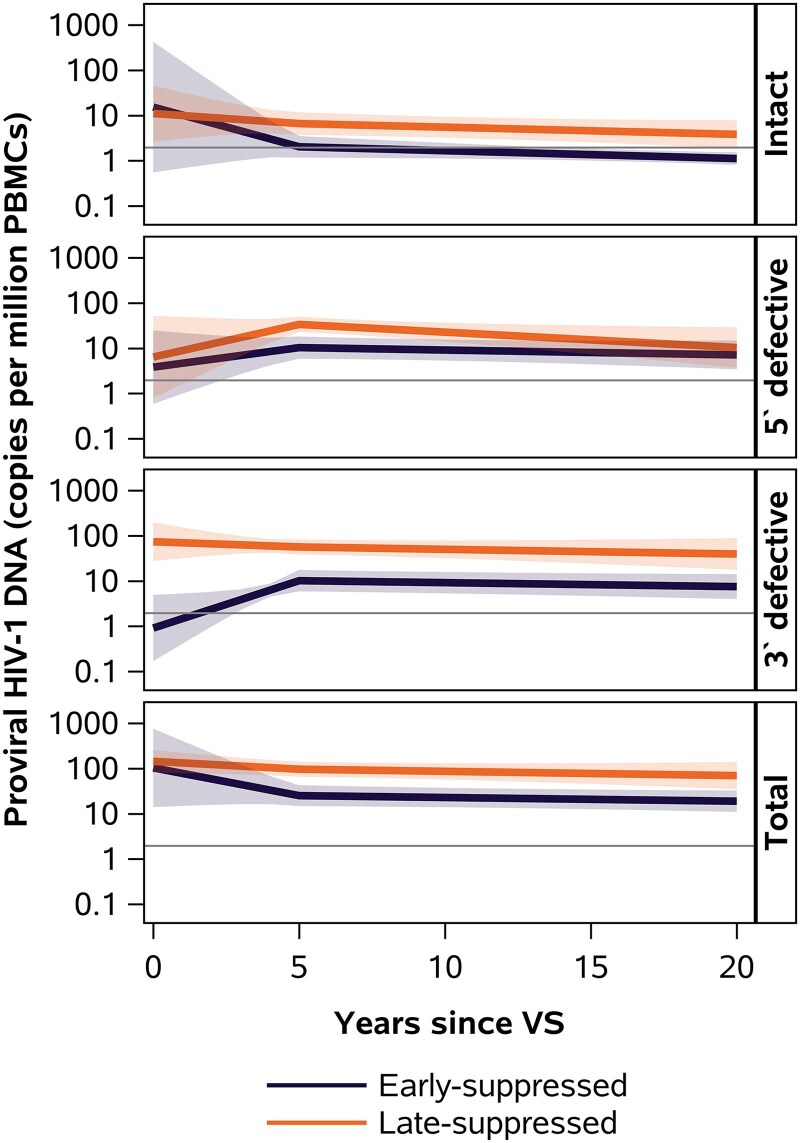
Mixed effects model estimated intact, 5′ defective, 3′ defective/hypermutated, and total proviral HIV DNA trajectories by age at virologic suppression. Predicted least squared means with 95% bands. Reference line set at the average limit of detection of 2 copies/million PBMCs. Abbreviations: PBMCs, peripheral blood mononuclear cells; VS, virologic suppression.

The estimated annual percent of change (95% CI) in intact proviruses for ES participants was −33.2% (95% CI: −64.2%, 24.7%) from 0 to 5 years, which decreased to −3.9% (95% CI: −7.6%, −0.1%) between 5 and 15 years of VS (Figure 2 and Table 3). Late-suppressed participants exhibited a similar but less steep trend with an initial decline in intact proviruses of −9.8% per year (95% CI: −31.7%–19.2%) over the first 5 years of VS and a −3.6% annual decline (95% CI: −7.9%–1.0%), from 5 to 15 years of VS. The estimated mean intact proviral load at 5 years of VS was 2.0 copies/million PBMCs in ES and 6.6 copies/million PBMCs in LS participants. By 15 years of VS, the estimated mean intact proviral load was below LOD at 1.4 copies/million PBMCs in ES but 4.6 copies/million PBMCs in LS participants (Table 3).

**Table 3.** Mixed Effects Model Estimated Proviral HIV DNA Trajectories and Population Means by Age at Virologic Suppression and Sex

Duration of VS (years)	Percent Change (95% CI) in HIV DNA Per Year		Estimated Mean (95% CI) HIV DNA cpm PBMCs		
	0–5 Years of VS	5–15 Years of VS	At VS	5 Years of VS	15 Years of VS
Intact					
Early suppressed (<1 year old at VS)	−33.2% (−64.2%, 24.7%)	−3.9% (−7.6%, −0.1%)	15.3 (0.6, 422.5)	2.0 (1.2, 3.5)	1.4 (1.0, 1.8)
Female	−10.7% (−21.6%, 1.8%)	−4.3% (−11.8%, 3.9%)	4.2 (2.5, 7.0)	2.4 (0.8, 6.8)	1.5 (0.9, 2.5)
Male	−56.9% (−84.4%, 18.6%)	−1.6% (−5.3%, 2.3%)	91.4 (0.5, 18573.9)	1.4 (0.9, 2.0)	1.2 (0.9, 1.6)
Late suppressed (1–5 years old at VS)	−9.8% (−31.7%, 19.2%)	−3.6% (−7.9%, 1.0%)	11.1 (2.7, 46.0)	6.6 (3.7, 11.8)	4.6 (2.5, 8.3)
Female	−15.2% (−39.6%, 19.1%)	−3.4% (−5.9%, −0.9%)	28.5 (3.4, 237.7)	12.5 (6.2, 25.1)	8.8 (3.8, 20.6)
Male	−27.8% (−36.1%, −18.4%)	−4.5% (−11.0%, 2.5%)	20.7 (9.4, 45.6)	4.1 (2.2, 7.7)	2.6 (1.5, 4.5)
5′ defective					
Early suppressed (<1 year old at VS)	22.0% (−13.0%, 71.0%)	−2.4% (−6.8%, 2.2%)	3.8 (0.6, 24.8)	10.3 (5.9, 18.2)	8.1 (4.4, 14.7)
Female	17.3% (2.3%, 34.6%)	4.0% (−2.3%, 10.7%)	4.2 (2.5, 6.9)	9.3 (3.2, 27.0)	13.7 (6.3, 29.7)
Male	75.1% (17.7%, 160.5%)	−7.9% (−10.4%, −5.3%)	0.8 (0.1, 6.3)	12.6 (9.3, 17.1)	5.6 (3.7, 8.4)
Late suppressed (1–5 years old at VS)	39.3% (−6.4%, 107.4%)	−7.6% (−13.5%, −1.1%)	6.4 (0.8, 52.0)	33.6 (22.8, 49.6)	15.3 (7.4, 31.8)
Female	18.1% (−20.6%, 75.5%)	1.9% (−2.0%, 5.8%)	15.2 (1.9, 123.6)	34.8 (15.3, 79.3)	41.8 (21.5, 81.5)
Male	−0.3% (−11.9%, 12.9%)	−13.3% (−21.0%, −4.8%)	28.3 (15.8, 50.7)	27.9 (20.1, 38.9)	6.7 (2.8, 16.0)
3′ defective/hypermutated					
Early suppressed (<1 year old at VS)	62.0% (6.9%, 145.6%)	−2.0% (−4.6%, 0.7%)	0.9 (0.2, 4.9)	10.2 (5.9, 17.5)	8.3 (4.7, 14.8)
Female	66.4% (32.1%, 109.7%)	−2.2% (−5.9%, 1.7%)	1.0 (0.8, 1.2)	12.9 (4.7, 35.7)	10.4 (4.2, 25.4)
Male	72.6% (−5.0%, 213.6%)	−2.5% (−5.9%, 1.0%)	0.6 (0.1, 7.4)	9.4 (5.7, 15.5)	7.3 (4.4, 12.2)
Late suppressed (1–5 years old at VS)	−5.2% (−23.5%, 17.5%)	−2.4% (−6.8%, 2.3%)	73.8 (27.7, 196.2)	56.5 (38.4, 83.0)	44.5 (24.2, 81.7)
Female	−1.1% (−15.2%, 15.3%)	3.1% (−0.5%, 6.9%)	73.4 (26.5, 203.6)	69.4 (30.3, 159.1)	94.5 (54.3, 164.4)
Male	−2.6% (−6.4%, 1.3%)	−5.4% (−11.7%, 1.5%)	48.8 (33.2, 71.6)	42.8 (29.6, 61.8)	24.6 (11.0, 54.9)
Total					
Early suppressed (<1 year old at VS)	−24.6% (−47.3%, 7.7%)	−1.9% (−4.7%, 1.1%)	103.5 (14.1, 760.8)	25.1 (14.8, 42.7)	20.8 (12.6, 34.3)
Female	7.3% (−5.6%, 22.0%)	−0.8% (−5.9%, 4.5%)	19.9 (10.0, 39.7)	28.3 (10.3, 77.6)	26.1 (11.5, 59.5)
Male	−36.0% (−60.4%, 3.2%)	−2.4% (−6.0%, 1.3%)	194.9 (16.8, 2255.5)	20.9 (14.1, 31.0)	16.4 (12.3, 21.8)
Late suppressed (1–5 years old at VS)	−7.7% (−15.4%, 0.8%)	−2.2% (−6.1%, 2.0%)	144.1 (80.5, 257.9)	96.6 (65.6, 142.1)	77.7 (45.7, 132.1)
Female	−11.4% (−21.1%, −0.6%)	2.4% (0.0%, 4.9%)	228.5 (99.5, 524.8)	124.8 (59.0, 263.9)	158.6 (92.5, 272.2)
Male	−10.5% (−14.7%, −6.1%)	−5.6% (−10.9%, −0.0%)	131.9 (89.6, 194.3)	75.8 (55.4, 103.9)	42.4 (23.5, 76.6)

### Dynamics of Defective and Total Proviral Load by Age at Virologic Suppression

Defective proviruses were also substantially lower in ES than LS participants (Figure 1 and Table 2). While defective proviruses increased slightly during the first 5 years of VS, there were no substantial trends in defective provirus concentrations over 5–20 years of VS in ES or LS participant samples (Figure 2 and Table 2).

Total proviral load was also consistently lower throughout VS among ES than LS participants (Figure 1 and Table 2). We observed faster decay rates for total proviral HIV DNA between 0 and 5 years of VS with slower decay rates after 5 years of VS, suggesting a natural slope change point about 5 years from the time of confirmed VS. We also investigated a purely statistical approach to identifying slope change points and found a slope change point at 5 years from the time of confirmed VS (Figure 2).

In mixed effects models, with the observed slope change point at 5 years of VS, the initial decline in total proviral load was steeper in the first 5 years of VS among ES compared to LS participants (Figure 2 and Table 3). This was followed by a more stable trajectory over the subsequent 5–15 years of VS.

### Differences in the Dynamics of Intact, Defective Proviruses and Total Proviral Load by Age at Virologic Suppression and Sex

**Figure 3.**
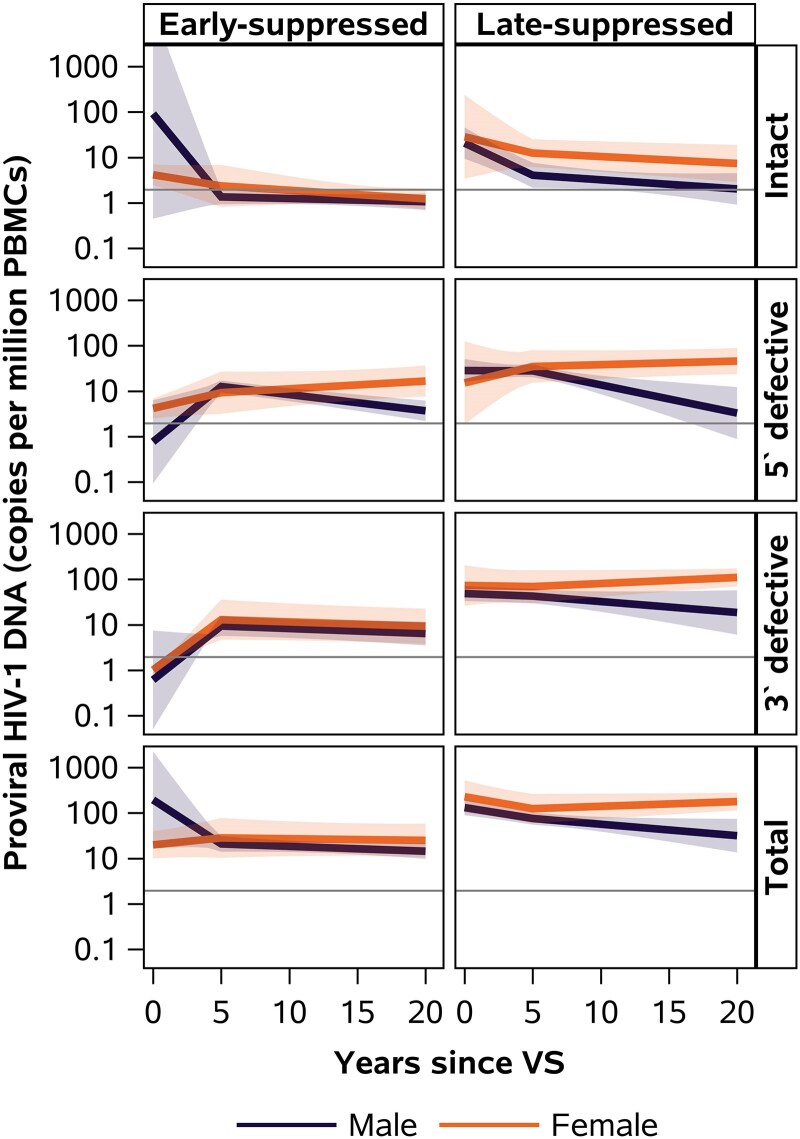
Mixed effects model estimated intact, 5′ defective, 3′ defective/hypermutated, and total proviral HIV DNA trajectories by age at virologic suppression and sex at birth. Predicted least squared means with 95% bands. Reference line set at the average limit of detection of 2 copies/million PBMCs. Abbreviations: PBMCs, peripheral blood mononuclear cells; VS, virologic suppression.

We could not effectively evaluate sex differences in intact proviral load in ES participants due to the high proportion of samples with undetectable intact proviruses (Table 2). Female LS participants, however, had higher median intact HIV DNA between 5 and 15 years of VS compared to male LS participants (Figure 3 and Table 3). While minimal differences in the trajectories of the intact proviruses were observed after 5 years of VS by sex among LS participants (Figure 3), estimated mean intact proviral loads were higher in females compared to males at 5 years of VS (12.5 vs 4.1 copies/million PBMCs) and 15 years of VS (8.8 vs 2.6 copies/million PBMCs; Table 3).

While we could not assess intact proviral load differences in ES participants, defective proviral loads also differed by sex in ES participants. Female ES participants had lower median levels of defective proviruses during the first 10 years of VS. However, after 10 years of VS, this difference shifted with female ES participants exhibiting higher median levels of defective proviruses than males (Supplementary Tables 1 and 2). In contrast, female LS participants had higher median levels of defective proviruses than males throughout the duration of VS. Notably, among LS participants, defective proviruses decreased in males but increased gradually in females during 5–15 years of VS (Figure 3). At 15 years of VS, the estimated mean defective proviral loads were higher in LS females than males (Table 3).

Across both ES and LS participants, females generally had higher total HIV DNA levels after 5 years of VS than males with larger differences observed among LS participants (Figure 3 and Table 3). Total HIV DNA among males LS participants decreased over 5–15 years of VS, while total HIV DNA gradually increased in females.

### Normalization for CD4+ T Counts Over Time

Although the IPDA in this study was performed on PBMCs, the HIV reservoir primarily resides in CD4+ T cells [17]. To account for differences in CD4+ T-cell counts with age and between participants that may obscure the true dynamics of the reservoir, IPDA assay results were normalized by the proportion of PBMCs that were CD4+ T cells. Interpretations of proviral subset dynamics were similar (Supplementary Figures 1–3).

## DISCUSSION

Understanding the dynamics of intact and defective proviruses in PHIV is critical for developing strategies to attain ART-free remission and cure for this population, which could avert a lifetime of ART for the estimated 1.4 million children with PHIV worldwide and additional 1 million adolescents with PHIV [1]. Recent studies from the PHACS cohort found that a child with PHIV in the United States has the potential to survive into the sixth and seventh decade of life and perhaps achieve normal life expectancy with optimized care [18].

We found that VS by 8 months of age led to markedly smaller reservoirs by 5 years of VS than with delayed VS, who achieved VS by 32 months of age. This difference emerged after the first 5 years of VS and persisted for 2 decades. Limited samples were available within the first 5 years of VS, preventing precise quantitation of intact proviruses early during VS. However, previous total HIV DNA quantitation of this cohort showed similar total HIV DNA levels at VS with a faster decline in the first 5 years of VS in the ES participants [16]. Early VS was also associated with fewer defective proviruses and lower total HIV DNA, supporting that early ART limits infected cell expansion in early childhood.

Negative indeterminate HIV serostatus is a consequence of early effective ART in PHIV [19]. In this same cohort of early-treated youth, we previously reported that 36% of 14 were HIV seronegative when tested at a median of age of 12.6 years, 11 of whom were included in this study, supporting our current findings of low intact proviruses with long-term early ART [20]. Multiple studies have shown that early ART by 3 months of age is associated with lower total HIV DNA in later childhood and adolescence, resulting in a distinct evolutionary trajectory of the proviral landscape in young adulthood [9, 21, 22].

Notably, with late VS, we observed that females had larger intact and defective provirus loads, and this difference was detected as early as by 5 years of VS, making hormonal effects of puberty less likely. Females with ES also exhibited this expansion of cells with defective proviruses after 10 years of VS. Although defective proviruses are unable to contribute to rebound thus not part of the viral reservoir, some retain the ability to express viral RNA and proteins, potentially contributing to immune dysregulation and inflammation [23]. The size, expansion, transcriptional, and translational activity of defective proviruses has not been examined in this cohort and requires further study.

Multiple studies globally show that children initiating ART in infancy have lower concentrations of infected cells in later life [21, 22, 24–38], underscoring the impact of age at VS on PHIV reservoirs. However, these studies were limited by lack of assessment of intact proviruses. Additionally, the effects of PHIV through young adulthood have not been fully studied. In our study, we were able to examine the dynamics of intact proviruses from infancy through young adulthood and found that intact proviral loads decayed faster within the first 5 years of VS before stabilizing. Similar early faster decay was noted in non-subtype B infections with neonatal ART in children in Botswana [24] and Kenyan [37]. Similarly, intact proviruses clear faster than defectives in adults, stabilizing after 5–7 years of ART [39]. Recent studies illustrate that extremely low intact proviral reservoirs can persist through the second decades of life in early-treated young adults with PHIV [21, 22]. The mechanisms underlying the transition from clearance to stability remain unclear, but may include HIV integration site distribution, capacity for viral expression, and immune-mediated processes such as increased NK-cell activity [22, 40].

Females LS participants had a 4-fold higher intact proviral load than males detected from 5 to 15 years of VS. This difference was also evident before puberty at 5–10 years of VS and persisted after adjusting for CD4+ T-cell percentages, suggesting immune differences rather than hormonal effects [41, 42]. Female children are known to have higher CD4+ T-cell counts [43–46], which would be consistent with the effects of normal CD4+ T-cell biology. We also observed larger total HIV DNA load and a higher proportion of defective proviruses in females, consistent with enhanced CD4+ T-cell survival or proliferation. Female ES participants showed increasing proportions of 5′ and 3′ defective/hypermutated proviruses over time, while males showed decreases. Our results are exploratory and whether this reflects sex-related immune differences will require further study [21, 46]. The high proportion of undetectable intact proviruses in the ES participants limited our ability to assess whether the intact reservoir was larger in females than males with early VS but can be examined in future studies.

Sex differences have been noted in untreated PHIV with females having lower viral loads and higher CD4+ T-cell counts than males [45, 46]. Recent studies found females exhibited increased susceptibility to in utero HIV [47] and greater risk of virologic failure following very early ART compared with males [32]. One study identified greater potential for ART-free remission in male children receiving very early ART [48], whereas this was not observed in the 4 cases of ART-free remission in a clinical trial [17]. Collectively, these data emphasize the importance of investigating sex differences in the immunopathogenesis of PHIV reservoirs to guide ART and immune-based therapies toward ART-free remission and cure.

### Limitations

Our study had several limitations. Our study sample was small but is reflective of the difficulty in maintaining viral suppression from infancy to young adulthood, for the majority of the population surviving with PHIV. Fewer participants had PBMC samples from 0 to 5 years and after 15 years of VS, limiting our ability to draw definitive conclusions about reservoir decay during these periods, although meaningful trends were observed. We a priori defined early suppression to extend through the first year of life due to the longer time to VS seen with early ART initiation by age 3 months [16, 49]. Another limitation is the use of PBMCs rather than purified CD4+ T cells, which reduced our capacity to quantify intact proviruses. Notably, in suppressed young adults, billions of cells were required to quantify intact proviruses [21, 22]. Our estimates of decay kinetics for these participants, particularly in later years of follow-up, are sensitive to our imputation of undetectable values to half the LOD. Future studies focusing on the first 5 years of VS with larger blood volumes in older age groups will be critical to further decipher proviral dynamics in PHIV and under newer and more contemporaneous therapies. Studies among young adults with long-term VS in settings outside of the United States are also needed.

Despite these limitations, our study evaluated the impact of early and delayed VS within similar birth cohorts and advances understanding of the early and long-term effects of ART on intact and defective proviral dynamics over decades of ART in PHIV, their persistence during VS, and the influence of age at VS and sex.

### Conclusion

With early long-term VS of PHIV, there is a paucity of intact proviruses following the first 5 years of VS. This contrasts with delayed VS, where intact proviruses remain readily detectable through 15 years of VS, emphasizing differences in proviral reservoir dynamics in PHIV as a function of age at and duration of ART. Sex differences should be considered in study design for reservoir assessment with implications for the timing and design of therapeutic strategies in this population and underscore the importance of early infant diagnosis and treatment.

## Supplementary Material

jiag045_Supplementary_Data

## Supplementary Data

Supplementary materials are available at *The Journal of Infectious Diseases* online (http://jid.oxfordjournals.org/). Supplementary materials consist of data provided by the author that are published to benefit the reader. The posted materials are not copyedited. The contents of all supplementary data are the sole responsibility of the authors. Questions or messages regarding errors should be addressed to the author.

## Notes

***Acknowledgments.*** We thank the participants and families for their participation in PHACS and the individuals and institutions involved in the conduct of PHACS. The graphical abstract was created in BioRender (https://BioRender.com). We thank Kristen Kelly for aiding with the graphical abstract and Soumia Bekka for helping with editing.

The following institutions, clinical site investigators, and staff participated in conducting PHACS AMP and AMP Up in 2022, in alphabetical order: *Ann & Robert H. Lurie Children's Hospital of Chicago*: Ellen Chadwick, Emanuela (Lela) Lartey, Rohit Kalra, Kathleen Malee; *Baylor College of Medicine*: Mary Paul, Shelley Buschur, Chivon McMullen-Jackson, Lynnette Harris; *BronxCare Health System*: Murli Purswani, Martha Cavallo, Mahboobullah Mirza Baig, Alma Villegas; *Children's Diagnostic & Treatment Center*: Lisa-Gaye Robinson, Alan Bernegger, Patricia Garvie; *Boston Children's Hospital*: Sandra K. Burchett, Michelle E. Anderson, Christine M. Salois; *Jacobi Medical Center*: Andrew Wiznia, Marlene Burey, Ray Shaw; *Rutgers—New Jersey Medical School*: Arry Dieudonne, Juliette Johnson, Karen Surowiec; *St. Christopher's Hospital for Children*: Janet S. Chen, Taesha White, Mitzie Grant; *St. Jude Children's Research Hospital*: Katherine Knapp, Erick Odero, Megan Wilkins; *San Juan Hospital Research Unit/Department of Pediatrics, San Juan Puerto Rico*: Nicolas Rosario, Heida Rios, Vivian Olivera; *Tulane University School of Medicine*: Margarita Silio, Medea Gabriel, Patricia Sirois; *University of California, San Diego*: Stephen A. Spector, Megan Loughran, Veronica Figueroa, Sharon Nichols; *University of Colorado Denver Health Sciences Center*: Elizabeth McFarland, Carrie Chambers, Christine Kwon, Robin McEnvoy; *University of Miami*: Gwendolyn Scott, Grace Alvarez, Juan Caffroni, Anai Cuadra

The Pediatric HIV/AIDS Cohort Study (PHACS) network was supported by the Eunice Kennedy Shriver National Institute of Child Health & Human Development (NICHD), Office of The Director, National Institutes of Health (OD), National Institute of Dental & Craniofacial Research (NIDCR), National Institute of Allergy and Infectious Diseases (NIAID), National Institute of Neurological Disorders and Stroke (NINDS), National Institute on Deafness and Other Communication Disorders (NIDCD), National Institute of Mental Health (NIMH), National Institute on Drug Abuse (NIDA), National Cancer Institute (NCI), National Institute on Alcohol Abuse and Alcoholism (NIAAA), and the National Heart, Lung, and Blood Institute (NHLBI) through grants to the Harvard T.H. Chan School of Public Health (P01HD103133, Principal Investigators: Ellen Chadwick, Sonia Hernandez-Diaz, Jennifer Jao, Paige Williams; Program Director: Liz Salomon and HD052102: Principal Investigator: George R Seage III; Program Director: Liz Salomon) and with Tulane University School of Medicine (HD052104) (Principal Investigator: Russell Van Dyke; Co-Principal Investigator: Ellen Chadwick; Project Director: Patrick Davis). Data management services were provided by Frontier Science (Data Management Center Director: Suzanne Siminski), and regulatory services and logistical support were provided by Westat, Inc. (Project Director: Tracy Wolbach).

***Author contributions.*** Research study design (D. P., K. P., P. K., and W. Y.), conducted experiments (P. K., J. S., A. D.), acquired data (K. P., W. Y., P. K., J. S., A. D., S. B., R. B. V. D.), conducted data analysis (K. P., W. Y., P. K., J. S., A. D.), supervised data analysis (K. P., D. P., and W. Y.), wrote the manuscript (K. P., D. P., A. D., P. K., W. Y.), reviewed and edited the manuscript (J. S., S. B., R. V. D.), and provided reagents and funding (D. P.).

***Study approval.*** PHACS AMP and AMP Up were approved by the institutional review board at the Harvard T.H. Chan School of Public Health and each participating site. Written informed consent was obtained from each participant or the participant's parent or legal guardian. Assent was obtained from child participants according to local institutional review board guidelines. The study was also approved by the Johns Hopkins University School of Medicine to process de-identified samples for laboratory studies quantifying HIV reservoirs in perinatal infection.

***Meeting(s) where this information was presented.*** CROI 2024, Denver CA.

***Data availability.*** The data described in the current article are not available at this time because of the confidential nature of the data and the current lack of a de-identified dataset. A limited dataset from PHACS AMP is available on the NICHD DASH system (https://dash.nichd.nih.gov/study/17510). Supporting analytic code may be made available upon reasonable request.

***Financial support.*** Research reported in this publication was supported by the National Institute of Allergy and Infectious Diseases of the National Institutes of Health under Award Number UM1AI164566 to DP for support of the Pediatric Adolescent Virus Elimination (PAVE) Martin Delaney Collaboratory. The content is soley the responsibility of the authors and does not necessarily represent the official views of the National Institutes of Health. D. P. is also supported by the National Institutes of Allergy and Infectious Diseases of the National Institutes of Health (AI150412, AI186792); the IMPAACT Lab Center (UM1AI106716); the HIV Prevention Trial Network 083 and 084 (NCT02720094 and NCT03164564); and the Johns Hopkins Center for AIDS Research (P30AI094189). The Pediatric HIV/AIDS Cohort Study (PHACS) network was supported by the Eunice Kennedy Shriver National Institute of Child Health & Human Development (NICHD), Office of The Director, National Institutes of Health (OD), National Institute of Dental & Craniofacial Research (NIDCR), National Institute of Allergy and Infectious Diseases (NIAID), National Institute of Neurological Disorders and Stroke (NINDS), National Institute on Deafness and Other Communication Disorders (NIDCD), National Institute of Mental Health (NIMH), National Institute on Drug Abuse (NIDA), National Cancer Institute (NCI), National Institute on Alcohol Abuse and Alcoholism (NIAAA), and the National Heart, Lung, and Blood Institute (NHLBI) through grants to the Harvard T.H. Chan School of Public Health (P01HD103133 and HD052102) and with Tulane University School of Medicine (HD052104).
