# Supplementary material for: Dynamics of Intact and Defective HIV-1 Proviruses during Decades of Suppressive Antiretroviral Treatment in Young Adults with Perinatal HIV-1
Source: J Infect Dis. Author manuscript; Available in PMC 2026 Mar 24. (PMC13007499; doi:10.1093/infdis/jiag045)
Supplement: Supplementary file 1 [file NIHMS2145250-supplement-Supplemental_Materials.docx]

**Supplemental Table** **1****: HIV-1 proviral DNA among all PBMC samples analyzed by follow-up period for FEMALE participants in the study population by age at virologic suppression.**

|  | ***All PBMC samples obtained among early suppressed (<1 year old) female participants (N=6)*** |  |  |  | ***All PBMC samples obtained among late suppressed (1-5 years old) female participants (N=7)*** |  |  |  |
| --- | --- | --- | --- | --- | --- | --- | --- | --- |
| ***HIV-1 DNA Species (copies/million PBMCs)***^***a***^ | ***0 to <5 of VS*** ***(N=7 samples from 3 participants)*** | ***5 to <10 of VS*** ***(N=11 samples from 5 participants)*** | ***10 to <15 of VS*** ***(N=12 samples from 5 participants)*** | ***15 to <20 of VS*** ***(N=12 samples from 5 participants)*** | ***0 to <5 of VS*** ***(N=11 samples from 5 participants)*** | ***5 to <10 of VS*** ***(N=14 samples from 6 participants)*** | ***10 to <15 of VS*** ***(N=25 samples from 7 participants)*** | ***15 to <20 of VS*** ***(N=6 samples from 4 participants)*** |
| Intact^b^ | 1.0 (0.8, 15.8) | 1.1 (0.9, 3.1) | 1.4 (1.1, 3.5) | 1.3 (1.1, 1.9) | 41.6 (4.1, 76.4) | *17.2 (8.4, 24.9)* | *13.4 (3.2, 24.1)* | 9.1 (3.7, 18.8) |
| *Detectable*^*b*^ | *2 (29%)* | *3 (27%)* | *5 (42%)* | *6 (50%)* | *10 (91%)* | *14 (100%)* | *20 (80%)* | *5 (83%)* |
| 5` defective^b^ | 3.3 (2.9, 6.6) | 3.3 (2.4, 15.4) | 18.9 (10.0, 42.2) | 24.6 (7.9, 41.1) | 75.6 (15.0, 94.7) | 67.4 (45.0, 81.2) | 54.9 (19.7, 73.4) | 69.3 (6.4, 94.9) |
| *Detectable*^*b*^ | *6 (86%)* | *10 (91%)* | *12 (100%)* | *12 (100%)* | *9 (82%)* | *14 (100%)* | *24 (96%)* | *6 (100%)* |
| 3` defective/ hypermutated^b^ | 3.2 (2.2, 11.6) | 4.8 (1.8, 15.4) | 29.3 (7.1, 55.7) | 13.5 (7.9, 30.4) | 76.8 (49.6, 138.9) | 140.5 (83.8, 182.6) | 111.4 (54.0, 145.1) | 129.9 (26.5, 196.8) |
| *Detectable*^*b*^ | *6 (86%)* | *9 (82%)* | *11 (92%)* | *12 (100%)* | *11 (100%)* | *14 (100%)* | *25 (100%)* | *6 (100%)* |
| Total^c^ | 12.2 (7.5, 17.3) | 8.1 (7.2, 32.1) | 54.7 (19.2, 113.2) | 39.8 (15.8, 74.2) | 217.3 (150.3, 372.8) | 210.8 (135.2, 288.4) | 188.8 (127.5, 220.7) | 213.0 (39.9, 297.8) |
| *Detectable*^*c*^ | *7 (100%)* | *11 (100%)* | *12 (100%)* | *12 (100%)* | *11 (100%)* | *14 (100%)* | *25 (100%)* | *6 (100%)* |
| ^a^ Summary statistics presented as either Median (Q1, Q3) or N (%).  ^b^ Values below the limit of detection imputed as half the limit of detection.  ^c^ HIV-1 DNA classified detectable if any of intact, 5` defective, or 3` defective/hypermutated HIV-1 DNA were above the limit of detection. Total HIV-1 DNA calculated as the sum of intact, 5` defective, and 3` defective/hypermutated HIV-1 DNA after imputation of values below the limit of detection.    PBMC: peripheral blood mononuclear cell; VS: virologic suppression |  |  |  |  |  |  |  |  |

**Supplemental Table** **2****: HIV-1 proviral DNA among all PBMC samples obtained by follow-up period for MALE participants in the study population by age at virologic suppression.**

|  | ***All PBMC samples obtained among early suppressed (<1 year old) male participants (N=5)*** |  |  |  | ***All PBMC samples obtained among late suppressed (1-5 years old) male participants (N=8)*** |  |  |  |
| --- | --- | --- | --- | --- | --- | --- | --- | --- |
| ***HIV-1 DNA Species (copies/million PBMCs)***^***a***^ | ***0 to <5 of VS*** ***(N=8 samples from 3 participants)*** | ***5 to <10 of VS*** ***(N=11 samples from 5 participants)*** | ***10 to <15 of VS*** ***(N=13 samples from 5 participants)*** | ***15 to <20 of VS*** ***(N=10 samples from 5 participants)*** | ***0 to <5 of VS*** ***(N=7 samples from 2 participants)*** | ***5 to <10 of VS*** ***(N=17 samples from 8 participants)*** | ***10 to <15 of VS*** ***(N=26 samples from 8 participants)*** | ***15 to <20 of VS*** ***(N=11 samples from 6 participants)*** |
| Intact^b^ | 1.1 (1.0, 85.2) | 1.4 (1.2, 3.2) | 1.0 (1.0, 1.1) | 0.9 (0.8, 1.1) | 12.4 (1.6, 25.0) | 4.1 (1.3, 6.4) | 3.2 (1.0, 8.2) | 1.3 (1.0, 11.5) |
| *Detectable*^*b*^ | *3 (38%)* | *4 (36%)* | *3 (23%)* | *2 (20%)* | *6 (86%)* | *11 (65%)* | *15 (58%)* | *7 (64%)* |
| 5` defective^b^ | 4.9 (1.6, 9.8) | 10.6 (8.5, 21.1) | 8.1 (3.1, 10.5) | 6.5 (4.2, 10.1) | 21.5 (17.2, 33.1) | 27.5 (13.3, 30.9) | 12.3 (4.3, 18.8) | 8.4 (1.6, 30.6) |
| *Detectable*^*b*^ | *6 (75%)* | *10 (91%)* | *13 (100%)* | *8 (80%)* | *7 (100%)* | *17 (100%)* | *22 (85%)* | *9 (82%)* |
| 3` defective/ hypermutated^b^ | 6.1 (1.1, 13.3) | 8.5 (3.6, 17.6) | 15.3 (6.4, 19.3) | 6.7 (4.8, 9.2) | 28.1 (14.1, 67.1) | 40.1 (19.1, 57.6) | 30.0 (15.5, 62.1) | 23.1 (5.7, 78.1) |
| *Detectable*^*b*^ | *5 (63%)* | *9 (82%)* | *12 (92%)* | *10 (100%)* | *7 (100%)* | *17 (100%)* | *25 (96%)* | *11 (100%)* |
| Total^c^ | 25.7 (16.6, 98.3) | 29.6 (19.9, 40.0) | 21.4 (18.8, 28.6) | 15.9 (11.4, 19.9) | 69.6 (35.3, 125.3) | 74.1 (40.2, 99.5) | 52.4 (27.6, 89.2) | 36.5 (17.6, 112.2) |
| *Detectable*^*c*^ | *8 (100%)* | *10 (91%)* | *13 (100%)* | *10 (100%)* | *7 (100%)* | *17 (100%)* | *26 (100%)* | *11 (100%)* |
| ^a^ Summary statistics presented as either Median (Q1, Q3) or N (%).  ^b^ Values below the limit of detection imputed as half the limit of detection.  ^c^ HIV-1 DNA classified detectable if any of intact, 5` defective, or 3` defective/hypermutated HIV-1 DNA were above the limit of detection. Total HIV-1 DNA calculated as the sum of intact, 5` defective, and 3` defective/hypermutated HIV-1 DNA after imputation of values below the limit of detection.    PBMC: peripheral blood mononuclear cell; VS: virologic suppression |  |  |  |  |  |  |  |  |

***Supplemental Figure 1******: LOESS of intact, 5`-defective, 3`-defective/hypermutated, and total HIV-1 DNA during virologic suppression, by age at virologic suppression (Y axis - per million CD4 cells)***

 

***Figure presents proviral HIV-1 DNA concentration in copies/million CD4 cells, by years since virologic suppression (VS), with LOESS trend lines overlaid atop actual data points. Unfilled circles represent values below the assay limit of detection which were imputed to be half the limit of detection.***

***Supplemental Figure 2******: Mixed effects model estimated intact, 5` defective, 3` defective/hypermutated, and total proviral HIV-1 DNA trajectories, by age at virologic suppression (Y axis - per million CD4 cells)***

 

***Predicted least squared means with 95% bands. Reference line set at average limit of detection of 5 copies/million CD4 cells***

***PBMC: peripheral blood mononuclear cells; VS: virologic suppression***

***Supplemental Figure 3******: Mixed effects model estimated intact, 5` defective, 3` defective/hypermutated, and total proviral HIV-1 DNA trajectories, by age at virologic suppression and sex at birth (Y axis - per million CD4 cells)***

 

***Predicted least squared means with 95% bands. Reference line set at average limit of detection of 5 copies/million CD4 cells***

***PBMC: peripheral blood mononuclear cells; VS: virologic suppression***
